# Mutations on the Switch III region and the alpha3 helix of Galpha_16 _differentially affect receptor coupling and regulation of downstream effectors

**DOI:** 10.1186/1750-2187-3-17

**Published:** 2008-11-22

**Authors:** May YM Yu, Maurice KC Ho, Andrew MF Liu, Yung H Wong

**Affiliations:** 1 Department of Biochemistry, Molecular Neuroscience Center and Biotechnology Research Institute, Hong Kong University of Science and Technology, Clear Water Bay, Kowloon, Hong Kong, PR China

## Abstract

**Background:**

Gα_16 _can activate phospholipase Cβ (PLCβ) directly like Gα_q_. It also couples to tetratricopeptide repeat 1 (TPR1) which is linked to Ras activation. It is unknown whether PLCβ and TPR1 interact with the same regions on Gα_16_. Previous studies on Gα_q _have defined two minimal clusters of amino acids that are essential for the coupling to PLCβ. Cognate residues in Gα_16 _might also be essential for interacting with PLCβ, and possibly contribute to TPR1 interaction and other signaling events.

**Results:**

Alanine mutations were introduced to the two amino acid clusters (246–248 and 259–260) in the switch III region and α3 helix of Gα_16_. Regulations of PLCβ and STAT3 were partially weakened by each cluster mutant. A mutant harboring mutations at both clusters generally produced stronger suppressions. Activation of Jun N-terminal kinase (JNK) by Gα_16 _was completely abolished by mutating either clusters. Contrastingly, phosphorylations of extracellular signal-regulated kinase (ERK) and nuclear factor κB (NF-κB) were not significantly affected by these mutations. The interactions between the mutants and PLCβ2 and TPR1 were also reduced in co-immunoprecipitation assays. Coupling between G_16 _and different categories of receptors was impaired by the mutations, with the effect of switch III mutations being more pronounced than those in the α3 helix. Mutations of both clusters almost completely abolished the receptor coupling and prevent receptor-induced Gβγ release.

**Conclusion:**

The integrity of the switch III region and α3 helix of Gα_16 _is critical for the activation of PLCβ, STAT3, and JNK but not ERK or NF-κB. Binding of Gα_16 _to PLCβ2 or TPR1 was reduced by the mutations of either cluster. The same region could also differentially affect the effectiveness of receptor coupling to G_16_. The studied region was shown to bear multiple functionally important roles of G_16_.

## Background

**Figure 1:**
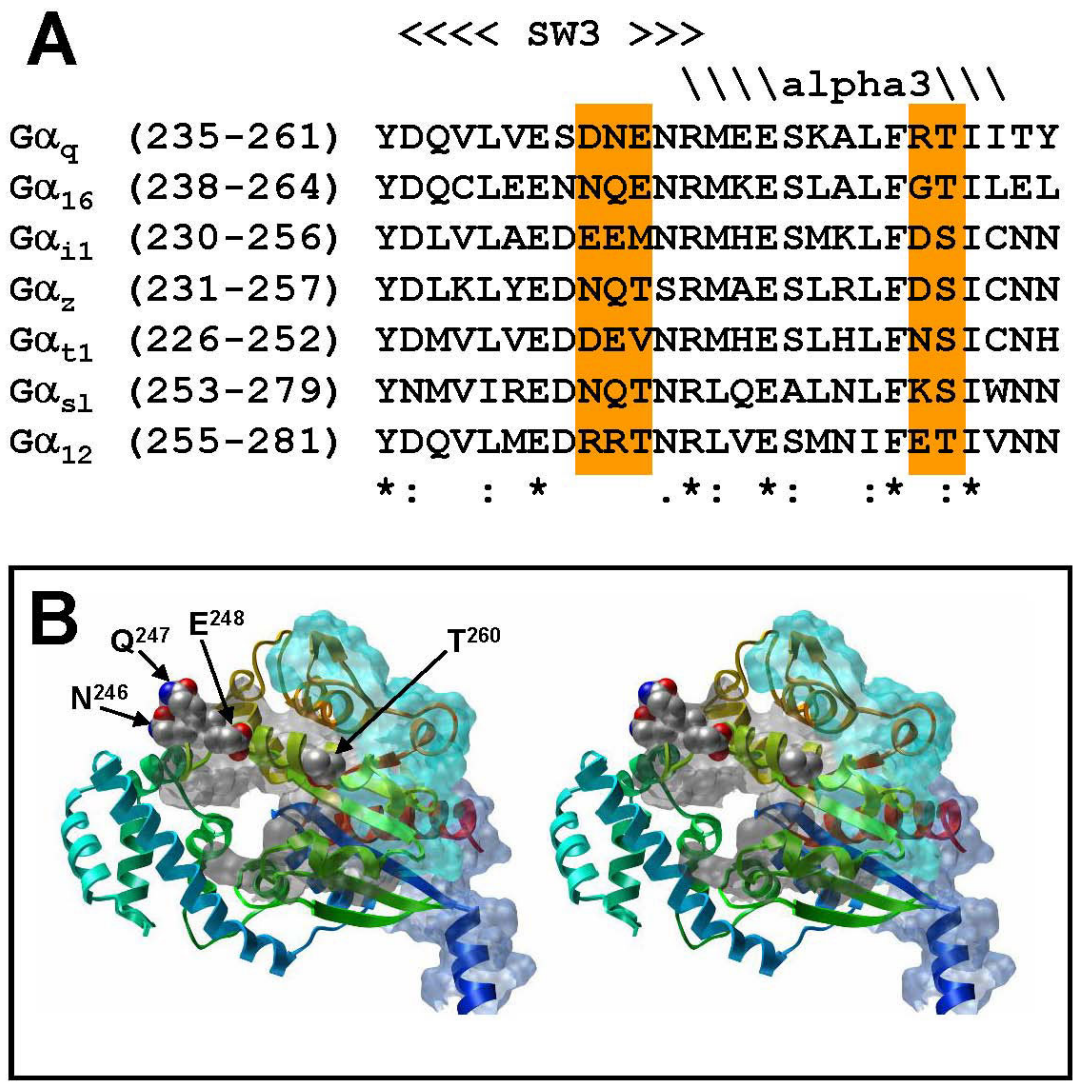
**Sequence alignment and molecular model of Gα_16_**.(A) The sequences corresponding to the switch III region and α3 helix of various Gα's were aligned. The consensus sequences are indicated as asterisks, colons and dots for strictly conserved, closely related and barely related residues among the candidates. The regions corresponding to the two clusters of putative PLC-interacting residues of Gα_q _are highlighted in orange. (B) A stereogram of the constructed molecular model of Gα_16 _is shown. Portions of the molecular surface were colored as blue, grey and cyan for the regions interacting with receptor, effector, or both, respectively, based on the studies of different G proteins. The side chains of the residues studied here are shown in spheres as indicated (except for Gly^259 ^which is devoid of any side chain).

As the major group of cell-surface detectors for hormones and neurotransmitters, G protein-coupled receptors (GPCRs) employ a variety of signal transduction pathways to regulate cellular functions. One of the primary signaling routes initiated upon activation of GPCRs is through the stimulation of PLCβ by members of the Gα_q _subfamily. PLCβ activity can in turn regulate many downstream kinases and transcription factors, thereby modulating cellular processes such as growth and differentiation. The interactions between PLCβ and Gα_q _subfamily members have been examined by mutagenesis studies. Alanine scanning mutagenesis of Gα_q _has identified a stretch of amino acids (Ile^217^-Lys^276^) that may be responsible for PLCβ interaction. Within this region, two groups of amino acids (Asp^243^, Asn^244^, Glu^245 ^and Arg^256^, Thr^257^; Figure 1A and 1B) have been suggested to be crucial for PLCβ interaction [1]. These two clusters of amino acids are located in the α3 helix and β4-α3 loop (Figure 1A) which exhibits dramatic conformational changes during G protein activation [2,3].

Gα_16 _is a member of G_q _subfamily which can activate PLCβ [4], and its unique promiscuity for GPCRs [4] highlights its importance in cellular signaling, especially in hematopoietic cells where it is restrictively expressed [5]. Recent studies have revealed that Gα_16_possesses additional signaling properties which may be independent of PLCβ activity. It has been demonstrated early on that interleukin-2 and interleukin-8 induce Gα_16_-mediated activation of ERK [6]. The use of a constitutively active mutant of Gα_16 _(Gα_16_QL) confirmed that it can indeed stimulate the activities of ERK [7] and JNK [8,9] in various cell types. Presumably these stimulatory signals proceed via PLCβ which triggers the cleavage of phosphatidylinositol bisphosphate to form IP_3 _and DAG, and the latter can modulate numerous signaling cascades through the activation of protein kinase C (PKC). The ability of Gα_16_QL to activate transcription factors such as STAT3 [7,10] and NF-κB [11,12] also requires PLCβ activity. The discovery of a novel binding partner of Gα_16_, tetratricopeptide repeat 1 (TPR1) [13] opens up new possibilities for the regulation of ERK and its downstream effectors. Since TPR1 prefers to bind to active Ras, its association with Gα_16 _may facilitate signaling along the Ras/Raf-1/MEK/ERK axis. However, no study has yet addressed the relative contributions of the PLCβ and TPR1 on the G_16_-mediated signaling events.

Although Gα_16 _shares only 55% identity to Gα_q _in the amino acid sequence, the two clusters of putative PLCβ-interacting residues can be found in the homologous regions of Gα_16_. Cluster 1 includes Asn^246^, Gln^247 ^and Glu^248^, while cluster 2 consists of Gly^259 ^and Thr^260^. It is noteworthy that both the glutamate and threonine at the end of each cluster are highly conserved among the G_q _subfamily members (Figure 1A). Mutations of these residues may impair PLCβ activation, and affect other downstream effectors which are dependent on PLCβ activity. Signals channeled through the TPR1/Ras route are unlikely to be affected by such mutations unless TPR1 and PLCβ interact with similar regions on Gα_16_. To date, the TPR1-interacting domain(s) of Gα_16 _has not been defined. Indeed, there are precedents for multifunctional domains in Gα subunits. The receptor- and effector-interacting regions (e.g., α2-β4, α3-β5, and α4-β6 loops) in Gα_s _and Gα_i _subunits have substantial overlaps ([14-20], also see Figure 1B). Therefore, it would be necessary to examine the possible impacts of these five residues on the ability of Gα_16 _to interact with different molecular partners. Furthermore, both the switch III region and α3 helix show substantial spatial rearrangements during the activation of a Gα subunit, mutations in these two regions may also perturb the receptor-mediated G protein activation.

In this study, the functional impacts of the two clusters of amino acids were investigated for receptor-dependent and independent regulation of different effectors of Gα_16_. The results suggested that the two regions of interest were important for the activation of PLCβ, STAT3, JNK but not ERK or NF-κB. Besides, interactions of Gα_16 _with TPR1 and PLCβ were also reduced in a similar fashion. The same regions could also differentially affect the effectiveness of receptor coupling to G_16_.

## Results

### Design and expression of the Gα_16 _mutants

**Figure 2:**
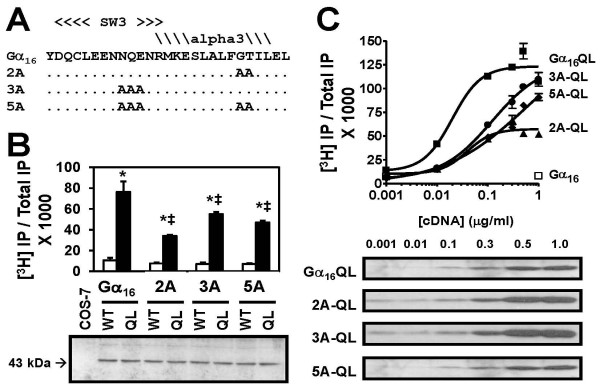
**Effects of 2A, 3A and 5A mutations on Gα_16_-mediated PLCβ activation**. (A) Positions of the alanine mutations on the corresponding Gα_16 _mutants were shown as an alignment with the Gα_16 _sequence. Identical residues were simplified with dots. (B) *Top: *COS-7 cells were transiently transfected with 0.25 μg/ml cDNAs encoding the wild type or QL mutants of Gα_16_, 2A, 3A and 5A. Transfectants were labeled with [^3^H]*myo*-inositol and assayed for IP accumulation. * IP accumulation stimulated by constitutively active mutants was significantly higher than that obtained with their wild type counterparts; ‡ Constitutive activity was significantly lower than that obtained with Gα_16_QL; Tukey-Kramer's test, p < 0.05. *Bottom: *Transfected COS-7 cells were harvested and membrane proteins were extracted for immunodetection. A Gα_16_-specific custom antiserum was used for recognition of Gα_16 _and its mutants. Fluorographs were visualized with the ECL chemiluminescence detection kit. Untransfected COS-7 cells served as the negative control. Two separate sets of transfected cells yielded similar results. (C) COS-7 cells were transfected with increasing amounts of cDNA encoding Gα_16_QL, 2A-QL, 3A-QL or 5A-QL. Empty vector pcDNA3 was added to balance the amount of cDNA used in the transfection for each sample. Gα_16_-transfected cells served as the negative control (hollow square). *Top: *IP production increased dose-dependently with increasing expression levels of the constitutively active form of alanine mutants and Gα_16_. *Bottom: *Expression level of constitutively active counterparts of Gα_16 _and its mutants were determined by Western blotting.

A previous study has shown that the residues lying on the switch III region and the α3 helix of Gα_q _are required for interaction with PLCβ [1]. The corresponding two clusters of amino acids on Gα_16 _are Asn^245^-Glu^247 ^and Gly^259^-Thr^260 ^(Figure 1A). Mutations of these two clusters of residues into alanine created 3 mutants (Figure 2A) denoted as 3A (Asn^245^-Glu^247 ^→ Ala), 2A (Gly^259^-Thr^260 ^→ Ala) and 5A (all 5 residues → Ala). The Gln^212 ^→ Leu (QL) mutation [8,21] was also introduced to individual alanine mutants to generate a constitutively active phenotype for studying effector interactions in a receptor-independent fashion. Previous studies have already confirmed the capability of Gα_16_QL to activate various downstream effectors such as ERK, NF-κB, STAT3 and JNK [7,12].

Since COS-7 cells do not express Gα_16 _endogenously, they are an excellent platform for studying Gα_16_-regulated signaling pathways by over-expressing Gα_16 _or its mutants. A tailor-designed antiserum [22] which recognizes the N-terminus (amino acid resides 13–27) of Gα_16 _was used for detecting the expression level of Gα_16 _and its mutants. Upon expression in COS-7 cells, a single band at around 43 kDa was detected by the anti-Gα_16 _antiserum for each construct (Figure 2B, lower part). Plasma membrane proteins prepared from non-transfected COS-7 cells served as a negative control (Figure 2B, leftmost lane). All of the mutants were expressed in COS-7 cells in a comparable level as Gα_16 _and Gα_16_QL, indicating that the mutations introduced did not affect the apparent expression or stability of Gα_16_.

### Impairment of PLCβ Regulation

The same sets of transfected cells examined in expression study were tested for their ability to stimulate PLCβ. Expression of Gα_16_QL in COS-7 cells significantly increased IP formation by ~7 fold as compared to cells expressing wild-type (WT) Gα_16_. Expression of constitutively active counterparts of the mutants (2A-QL, 3A-QL and 5A-QL) in COS-7 cells significantly increased IP accumulation as compared to the responses of their corresponding non-QL counterparts. Responses induced by QL forms of the mutants were significantly reduced as compared with the response elicited by Gα_16_QL (Figure 2B). 2A-QL and 3A-QL retained 42% and 73% of the IP production of Gα_16_QL, respectively, whereas 5A-QL preserved about 61% of the IP accumulation of Gα_16_QL. However, the differences between the mutants were not statistically significant. Apparently, the results indicated that the mutations partially impaired the ability of Gα_16 _to activate PLCβ.

Further experiments were carried out to verify the observed variations of the induced IP formation between different constitutively active mutants. Increasing amounts of individual QL mutant cDNA were transfected into COS-7 cells and the IP accumulation in the transfected cells were measured. For all of the QL mutants, increasing levels of IP accumulation were observed as the cDNA amounts were increased (Figure 2C, upper part). The expression levels of individual QL mutants were correspondingly increased as examined by Western blotting (Figure 2C, lower part). There were obvious differences in the PLCβ responses induced by the different mutants. Gα_16_QL and 2A-QL induced maximal stimulation of IP production at ~0.1 μg/ml, while maximal responses for 3A-QL and 5A-QL were only obtained at cDNA concentrations of 0.5 μg/ml or higher. Despite similar levels of expression, the maximal response stimulated by 2A-QL was only about half of that elicited by Gα_16_QL. The estimated EC_50 _values of IP accumulation were 0.020, 0.039, 0.123 and 0.610 μg/ml for Gα_16_QL, 2A-QL, 3A-QL and 5A-QL, respectively. These results indicate a progressive decrease of the efficiencies for activating PLCβ when one or more of the putative PLCβ-interacting domains on Gα_16 _were mutated to alanine. Mutations at the α3 helix (2A) might be particularly detrimental to PLCβ activity, because the maximal stimulatory response of 2A-QL was always lower than those of Gα_16_QL and the other two alanine mutants. Since 5A-QL could attain a higher maximal PLCβ response than 2A-QL, the incorporation of 3A mutations in the switch III region apparently relieved the functional impairment associated with the 2A mutations in the α3 helix of Gα_16_.

### Differential Regulations of Downstream Effectors

**Figure 3:**
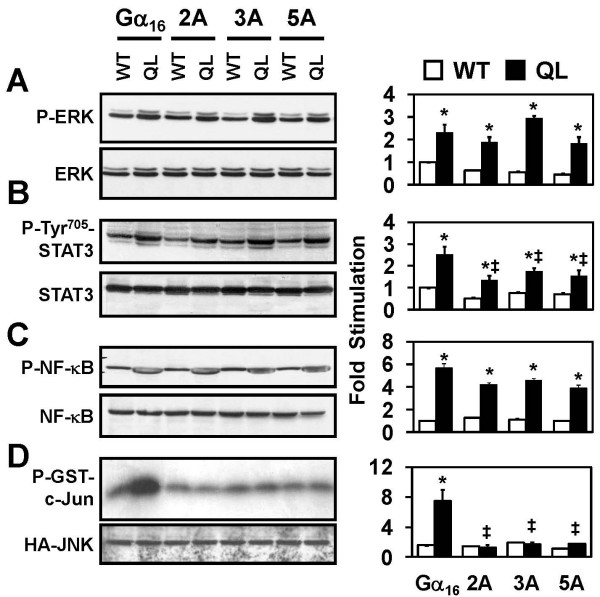
**Role of Gα _16 _mutants in STAT3, ERK1/2, NF-κB and c-Jun phosphorylation**. HEK293 cells were transfected with pcDNA3, wild type or QL mutants of Gα_16_, 2A, 3A or 5A. The transfectants were deprived of serum overnight, and cell lysates were prepared for SDS-PAGE separation. Phosphorylated form or native ERK1/2 (A), STAT3 (B), and NF-κB (C) were detected by Western blotting as indicated. (D) For measuring Gα_16_-triggered JNK activity, COS-7 cells were transiently transfected with each of the constructs mentioned together with a plasmid encoding HA-tagged JNK. Serum starvation was performed as mentioned and JNK assay was performed as described in Methods. Expression of tagged JNK was determined by anti-HA antibody. The activation of JNK was monitored by detecting the phosphorylation of GST-fused-cJun. The fold induction of the phosphorylation of various effectors were quantified and plotted on the right hand side for comparisons. * QL counterparts of the mutants stimulate phosphorylation of the detected proteins significantly over cells expressing wild type complements (Tukey-Kramer's test, p < 0.05). ‡ QL counterparts of 2A-, 3A- and 5A-stimulated phosphorylation were significantly lower than that of Gα_16_QL (Tukey-Kramer's test, p < 0.05).

Diverse downstream effectors have been found to be regulated by Gα_16_, including ERK, STAT3, NF-κB and JNK [7,9,11,23], but their dependencies on PLCβ activation have not been well defined. To further investigate the functions of the selected amino acids in regulating these downstream effectors, WT and QL forms of Gα_16 _and the three mutants were examined for their regulations of these downstream effectors when expressed in HEK293 cells. We employed HEK293 cells in this part of the study because of the regulations of ERK, STAT3, and NF-κB had been fully characterized in this cellular background [7,11] and all of the mutants could be efficiently expressed in these cells (data not shown). As illustrated in Figure 3A, no observable alteration in the total ERK1/2 levels was found in cells transfected with different cDNA constructs. The induction of ERK phosphorylation by the QL forms of the alanine mutants was significantly higher than their corresponding WT controls, and they were all similar to Gα_16_QL. It should be noted that 2A-QL and 5A-QL consistently generated a slightly lower level of ERK phosphorylation than 3A-QL and Gα_16_QL.

As both PLCβ and ERK can serve as upstream regulators of STAT3 [7], we examined the ability of the mutants to induce STAT3 Tyr^705 ^phosphorylation. All three mutants were capable of stimulating STAT3 phosphorylation at Tyr^705 ^albeit weaker than that induced by Gα_16_QL (Figure 3B). The phosphorylation triggered by 2A-QL, 3A-QL and 5A-QL were not significantly different between each other. Given that Gα_16_QL-induced STAT3 phosphorylation involves PLCβ and PKC [7], these results were not surprising because the alanine mutants have impaired PLCβ regulation (Figure 2).

Regulation of NF-κB by Gα_16 _has been demonstrated in recombinant systems as well as in human lymphoblastoma REH cells [11]. The phosphorylation of NF-κB induced by constitutively active alanine mutants was examined. As compared to Gα_16_, the expression levels of total NF-κB were unaffected in cells transfected with the alanine mutants (Figure 3C). The levels of NF-κB phosphorylation caused by the constitutively active alanine mutants were only slightly reduced (but not statistically different) when compared to the Gα_16_QL responses. There was also no obvious difference between 2A, 3A and 5A mutants.

Since the background phosphorylation of JNK in transfected HEK293 cells are relatively high (Chan AS and Wong YH, unpublished observation), *in vitro *kinase assay was adopted to determine the activity of JNK activity in COS-7 cells instead. COS-7 cells were co-transfected with Gα_16 _mutants together with HA-tagged JNK. The expression level of HA-tagged JNK were similar in all transfected cells (Figure 3D). Gα_16_QL enhanced JNK-mediated [^32^P]-labeling of GST-c-Jun significantly by 3.6-fold as compared to that of Gα_16_. Surprisingly, none of the constitutively active alanine mutants was capable of inducing JNK activation (Figure 3D). It appeared that the sequence integrity on the switch III region and α 3 helix is more critical for the regulation of JNK activation.

### Differential Associations with PLCβ and TPR1

**Figure 4:**
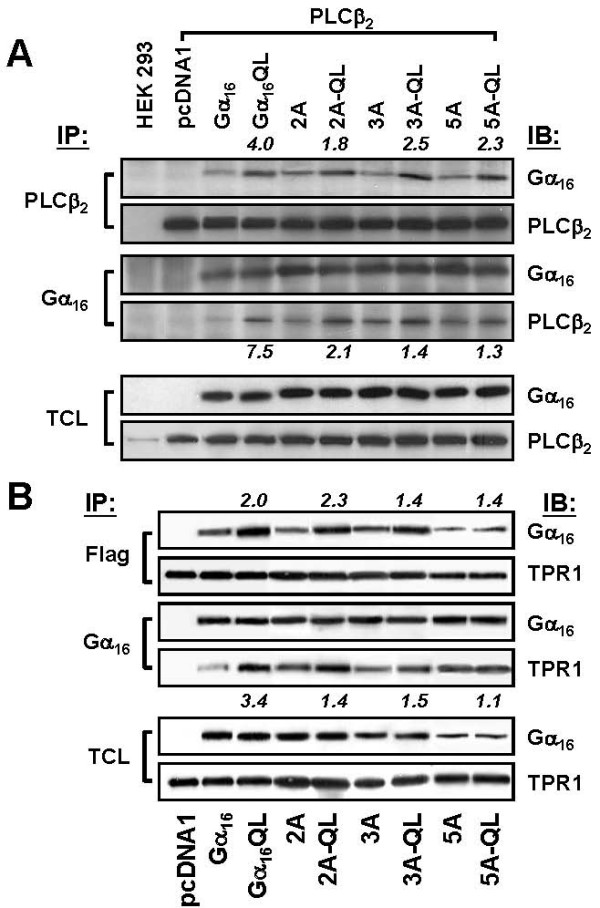
**The alanine mutants co-immunoprecipitate with TPR1 and PLCβ2**. (A) HEK293 cells were co-transfected with PLCβ2 and the Gα constructs as indicated at the bottom of the blots. Total cell lysates (TCL) from each condition were subjected to immunoprecipitation using either anti-PLCβ2 or anti-Gα_16 _antiserum followed by protein G-agarose. Well-washed immunoprecipitates were subjected to SDS-PAGE and the proteins of interest were detected using specific antibodies as indicated. TCL were separately run on blots for detecting the corresponding protein expressions. Two separate sets of transfected cells yielded similar results. (B) Similar procedures were applied for detecting the interaction between Flag-tagged TPR1 and the Gα constructs as indicated. TCL were subjected to immunoprecipitation using either anti-Flag agarose gel or anti-Gα_16 _antiserum followed by protein G-agarose. Two separate sets of transfected cells yielded similar results. Band intensities were quantified and figures on the lanes of QL mutants are the fold increase compared with the band on the lane of their corresponding wild-type counterparts.

As the alanine mutations of the two clusters caused reduction of PLCβ activation, one of the possible explanations would be the impairment of cognate recognition of PLCβ. Co-immunoprecipitation experiments were performed to study the possible alteration of the mutants and PLCβ2, a PLCβ isoform commonly expressed and known to interact with different G_q _family members (Figure 4A). Expression levels of different Gα_16 _constructs were similar when co-expressed with recombinant PLCβ2, as revealed in the total cell lysates. The endogenously expressed PLCβ2 could be detected (lane 1 of the bottom strip in Figure 4A) but the level was too low for co-immunoprecipitation assays. Both Gα_16 _and Gα_16_QL could be precipitated with PLCβ2 using either anti-Gα_16 _or anti-PLCβ2 antibodies, while Gα_16_QL showed much stronger interaction, indicating that the activation of Gα_16 _facilitated its interaction with PLCβ2. Similarly, the QL forms of either one of the three mutants showed better interactions with PLCβ2 when compared with their corresponding wild-type forms. However, the fold increases between the three alanine mutants and their corresponding QL forms were much lower than the Gα_16 _pair (values on top of each lane as indicated in Figure 4A). Such reduction was apparently due to the slight enhancement of the interactions between the alanine mutants and PLCβ2 as compared with Gα_16_. No significant difference of the fold changes was observed between the three mutant pairs. Nonetheless, the results clearly indicated that the Gα_16_/PLCβ interaction was impaired by both clusters of alanine mutations.

One of the distinctive features of Gα_16_-mediated signaling is the association with TPR1, which facilitates the accumulation of active Ras [13] and in turn activate the Raf/MEK/ERK cascade. To study the impact of the alanine mutations on the interaction between Gα_16 _and TPR1, a Flag-tagged full-length TPR1 was co-expressed with each of the Gα_16 _constructs as indicated in Figure 4. Co-immunoprecipitation of the proteins of interest was performed using anti-Flag tag or anti-Gα_16 _antibodies. Both Gα_16 _and Gα_16_QL could be co-immunoprecipitated with TPR1 (Figure 4); Gα_16_QL was bound to TPR1 more efficiently under our experimental condition. A truncated form of TPR1 lacking the C-terminal tail did not bind to Gα_16 _(Rico K. Lo, Andrew M. Liu and Yung H. Wong, data not shown). The three alanine mutants could also be co-immunoprecipitated with TPR1. Similar to the Gα_16_-PLCβ2 interactions, the QL versions of the alanine mutants showed stronger interactions with TPR1 than their corresponding non-active counterparts. Fold enhancements between the QL form of the alanine mutants and the non-active forms were weaker than Gα_16 _pair. The 5A-QL mutant showed the weakest enhancement of TPR1 interaction. Expression levels of 5A and 5A-QL might be less in the experiment as showed in the total cell lysates, but the fold enhancement was still the least among the others. More obvious results were obtained when the TPR1-Gα_16 _complexes were immunoprecipitated with anti-Gα_16 _antibody. The results suggested that the interactions between TPR1 and Gα_16 _were also dependent on the identities of the two amino acid clusters. Alanine mutations at both clusters caused the greatest reduction of the interaction.

### Perturbation of Receptor-Mediated Regulation of PLCβ

G_16 _is well-known for its receptor coupling promiscuity [4], but it also exhibits different degrees of coupling efficiencies to various GPCRs [22,24]. Since the receptor- and effector-interacting domains overlap partially on the surface of Gα subunits, it is possible that the alanine mutations can also affect the receptor coupling of Gα_16_. To test this possibility, a panel of GPCRs was examined for their functional coupling to Gα_16 _and the alanine mutants using the IP accumulation assay. These GPCRs included G_i_-coupled adenosine A_1 _receptor (A_1_R), complement C5aR receptor, formyl peptide receptor (fMLPR), and the G_s_-coupled adenosine A_2A _and A_2B _receptors (A_2A_R, A_2B_R) and dopamine D_1 _receptor (D_1_R). All of these receptors are capable of utilizing Gα_16 _to elicit intracellular calcium mobilization in FLIPR assays [24].

**Figure 5:**
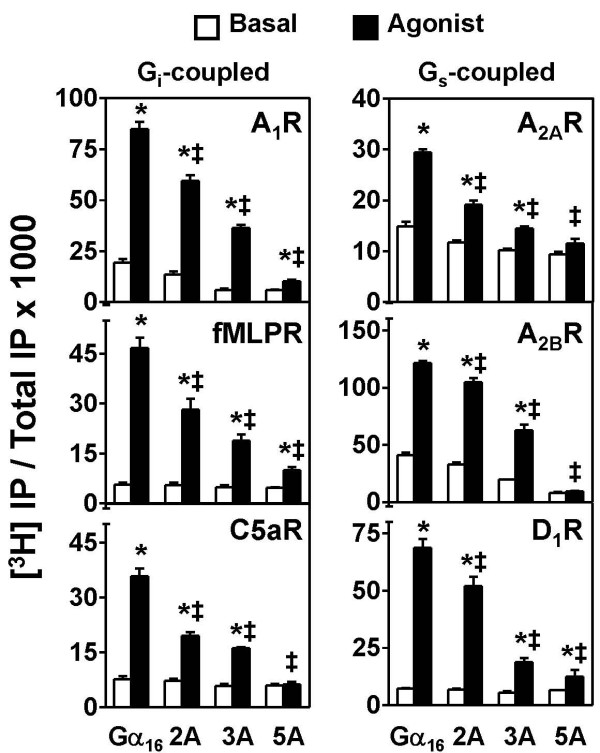
**Coupling of alanine mutants of Gα _16 _to different categories of G protein-coupled receptors**. Individual GPCRs were coexpressed with Gα_16_, 2A, 3A or 5A in COS-7 cells. Transfected cells were labeled with [^3^H]*myo*-inositol and treated with PTX overnight. Cells were then treated with the appropriate receptor agonists (1 μM CHA for A_1_R, 100 nM C5a for C5aR, 100 nM fMLP for fMLPR, 10 μM adenosine for both A_2A_R and A_2B_R, 10 μM dopamine for D_1_R) for an hour before extraction of accumulated labeled IP. *Agonist significantly stimulated IP production as compared to their corresponding untreated counterparts; Tukey-Kramer's test, p < 0.05. ‡ Agonist-induced responses in mutant-expressing cells were significantly lower than that obtained with Gα_16_; Tukey-Kramer's test, p < 0.05.

The selected G_i_- or G_s_-coupled receptors all induced IP accumulation significantly in the presence of Gα_16_, but with variable efficiencies (Figure 5). These receptors were also capable of interacting with the alanine mutants. In general, IP accumulation mediated by 2A and 3A were ~50% and 35%, respectively, of that induced by Gα_16_. In some cases, such as C5aR and A_2A_R, the differences between the responses of 2A and 3A mutants were not significant, but it may be due to the weaker overall responses when compared with other receptors. Contrastingly, the differences between the phenotypes of these two mutants in the coupling to A_1_R, A_2B_R and D_1_R were more obvious (≥ 50%). Differences in the absolute responses of various receptors have been demonstrated previously in the measurement of intracellular Ca^2+ ^mobilization (see Table 1 of [24]). The differential coupling efficiencies of the tested GPCRs with G_16 _allowed us to detect the differences on GPCR coupling between the alanine mutants. Agonist-induced activation of 5A could only marginally increase IP accumulation in cases of A_1_R, fMLPR and D_1_R, while the other receptors were totally unable to elicit a response via 5A (Figure 5). The defective phenotype indicated that the two clusters were essential for the effective receptor-mediated PLCβ activation through Gα_16_. The switch III region (harboring the 3A mutations) appeared to be more influential to the productive receptor coupling events than the α 3 helix.

Upon closer examination of the basal levels of the cells expressing Gα_16 _or the 3 mutants, it was noticed that the basal levels of IP accumulation in the cells expressing A_1_R, A_2A_R or A_2B_R with Gα_16 _were higher than with the mutants (Figure 5). This trend of decreasing basal IP accumulation was similar to the receptor-activated responses, with Gα_16_>2A>3A>5A. The results here showed that the basal activities of all three adenosine receptors were suppressed by the expression of the alanine mutants, particularly for 5A. Such evidence further implied that the alanine mutants might associate with the receptors, but such receptor-G protein complexes were defective and the mutants actually sequestered the spontaneous activities of the receptors (also see Discussion).

### 5A Mutant was Defective in Receptor-Mediated Regulation of Type II Adenylyl Cyclase

**Figure 6:**
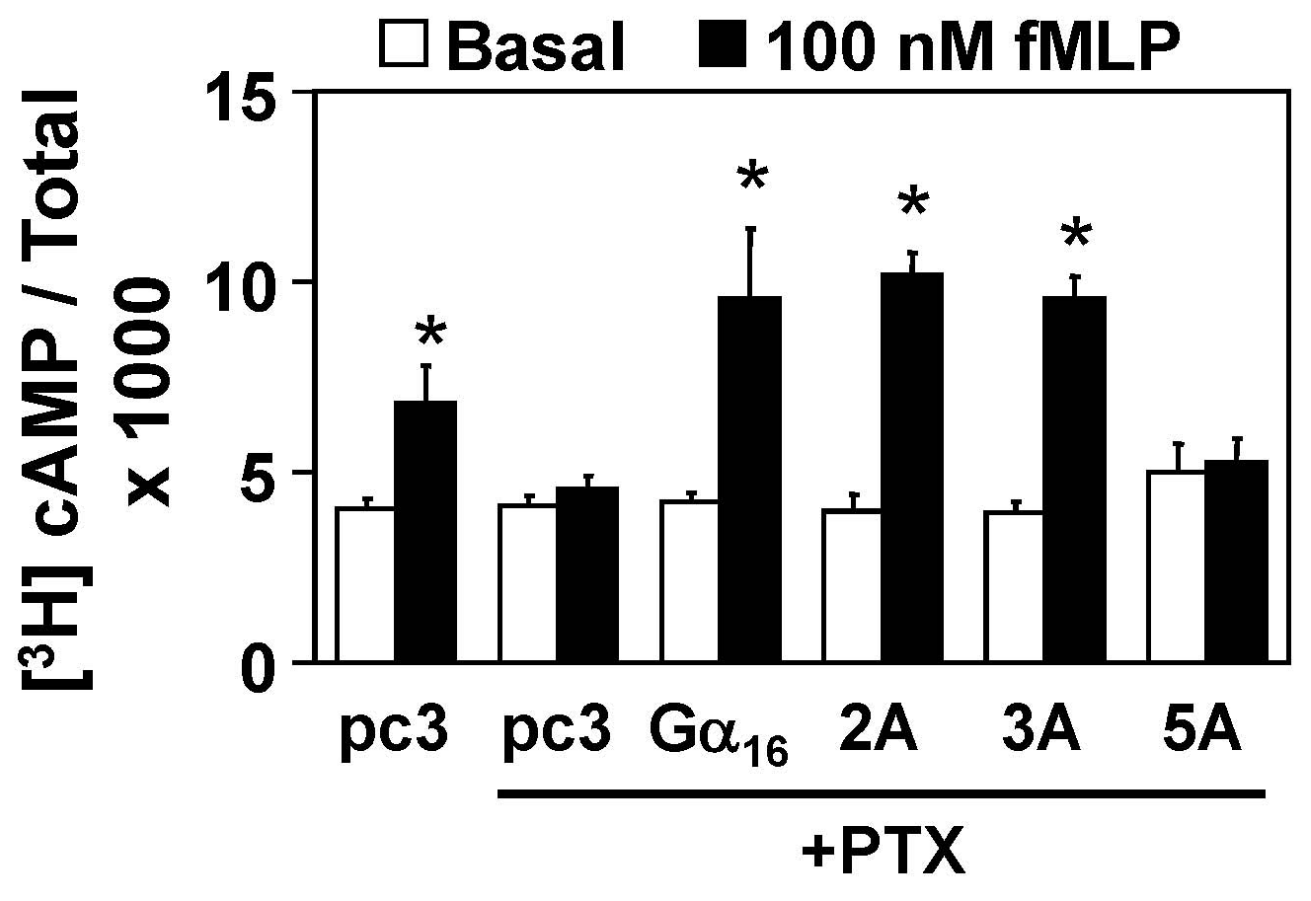
**Receptor-induced activation of type II adenylyl cyclase (AC2) mediated by Gα _16 _and its mutants**. HEK293 cells were cotransfected with cDNAs encoding the AC2 (3 μg/ml), Gα_s_QL (0.015 μg/ml) and fMLPR (3 μg/ml) together with Gα_16_, 2A, 3A or 5A. Cells were labeled with [^3^H]adenine and treated with or without PTX (100 ng/ml) overnight. cAMP accumulation was assayed in response to the treatment with 100 nM fMLP for 1 h. * cAMP accumulation was significantly increased as compared with their corresponding basal value; Tukey-Kramer's test, p < 0.05.

Type 2 adenylyl cyclase (AC2) can be synergistically stimulated via the Gβγ complex released from Gα in the presence of activated Gα_s _[25,26]. Measurement of AC2-mediated cAMP production in such circumstance is useful for the detection of receptor-mediated release of Gβγ subunits. Functional interaction of the alanine mutants with G_i_-coupled receptors, if any, would be expected to stimulate AC2 upon agonist triggering. HEK293 cells were co-transfected with empty vector pcDNA3 and cDNAs encoding Gα_s_QL, fMLPR and AC2. Activation of the fMLPR resulted in the stimulation of endogenous G_i _and release Gβγ which led to the activation of AC2 (Figure 6). PTX pretreatment attenuated the corresponding increase of cAMP level. In cells co-expressing Gα_16_, Gα_s_QL, fMLPR and AC2, activation of fMLPR led to an increase in cAMP formation in a PTX-insensitive manner (Figure 6), indicating the functional coupling of fMLPR with Gα_16_. Both 2A and 3A mutants exhibited comparable significant increases in cAMP production as Gα_16_. However, 5A was totally incapable of stimulating AC2, which suggested that the receptor-mediated activation of G_16 _and the subsequent Gβγ release might be severely impaired by the mutations on both switch III and α 3 helix.

## Discussion

There are at least two paths through which Gα_16 _can transmit signals to downstream effectors, via PLCβ or TPR1. Their relative contributions to well-established downstream effectors such as ERK, STAT3, NF-κB and JNK have not been clearly addressed. This study attempted to decipher the differential roles of the two streams of signals by mutating putative "PLCβ-interacting" residues on switch III and α 3 helix of Gα_16_. All three mutants (2A, 3A, and 5A) exhibited impaired ability to stimulate PLCβ. Differential regulations of ERK, STAT3, NF-κB and JNK by the mutants have been observed, suggesting that Gα_16 _regulates these downstream signals through overlapping but discrete pathways. Interactions between the mutants and PLCβ2 or TPR1 were reduced by the alanine mutations, and mutations of both clusters caused the greatest reduction of interactions. Furthermore, the 5A mutant with both clusters mutated was severely impaired in receptor coupling, as was observed in receptor-mediated activation of PLCβ and AC2, and the sequestration of constitutive receptor activity. Collectively, this study provided evidence that the integrity of the switch III region and α 3 helix of Gα_16 _was critical for both PLCβ-and TPR1-dependent signaling events.

Gα_q _and Gα_16 _belong to the same subfamily of heterotrimeric Gα subunits and both regulate PLCβ in a similar fashion. Presumably, the molecular interactions between Gα_q _and Gα_16 _with PLCβ would share some common features. Based on the previous study on Gα_q _[1], the five homologous residues on Gα_16 _were mutated into alanine, and the resultant mutants showed partial impairment on PLCβ regulation (Figures 2). Employment of Q212L mutation bypassed the possible influence on receptor coupling as well as the effect of Gβγ complex release, which may also activate PLCβ in its own. The incomplete impairment suggests that the Gα-effector interactions may involve multiple contact sites, as documented in the studies on other Gα subunits [15,17,18]. Co-immunoprecipitation experiments showed that the physical association between PLCβ and Gα_16 _was not completely abolished by the mutations of either clusters (Figure 4). The mere association between the two proteins was apparently not sufficient to exert the full activating effect. Mutants bearing the 2A mutations on the surface of α 3 helix of Gα_16 _were less effective in PLC activation (Figure 2). These two residues were more likely to contribute to the direct interaction and activation of PLCβ, and so have a greater effect. The switch III region where 3A mutations were located has been suggested to influence the interdomain interactions between the helical and GTPase domain of Gα_s _subunit, which in turn affect the GTP-induced activation [27]. Assuming the activation mechanisms of the homologous Gα subunits were very similar, the weaker suppression on PLCβ activation by 3A mutations might be an indirect effect. As all of the mutants were expressed to a comparable level (Figure 2), the differences in PLCβ activation could not be attributed to insufficient expression of a particular mutant.

We have previously proposed that Gα_16_QL-induced STAT3 phosphorylation is mediated via c-Src/JAK and ERK pathways, with PLCβ/PKC serving as upstream modulators [7,11]. Reduction of PLCβ activation caused by the mutations may disturb the signaling cascades at multiple points, and the modulation of downstream effectors may be altered differentially depending on the strength of upstream signals to be integrated. The present study shows that the alanine mutants partially inhibited the phosphorylation of STAT3 (Figure 3B), and the inhibitory profiles are reminiscent of the PLCβ activities (Figure 2B). However, the same mutants had very little effect on ERK phosphorylation (Figure 3A). These results suggest that Gα_16_QL-induced PLCβ signal is important for STAT3 phosphorylation, but not ERK phosphorylation. With the discovery of TPR1, Gα_16 _may stimulate the Raf/MEK/ERK axis through Ras. However, Ras-mediated activation of ERK is not the only ERK-regulating signal. Other streams of signals contributing to ERK activation include the PLCβ-mediated Ca^2+ ^mobilization and PKC activation, as well as possibly c-Src and JAK [10,11]. Furthermore, various molecular scaffolds for ERK pathway intermediates, GRKs and other signaling components may also contribute to the specificity of the ERK regulation [28,29] induced by the activation of Gα_16_, which have not been rigorously studied here. The dependence of STAT3 activity on ERK is somewhat controversial, because ERK has been shown to negatively regulate STAT3 [30]. JNK, on the other hand, appears to be necessary for Src-mediated activation of STAT3 [31]. In this regard, it is interesting to note that all three mutants exhibited defective regulations of JNK (Figure 3D). It remains to be determined if JNK/Src signals are critical for the regulation of STAT3 tyrosine phosphorylation.

Gα_16_QL is known to activate JNK in various cell types [8,9]. The complete loss of the ability of all three alanine mutants to stimulate JNK suggests that Gα_16_-induced PLCβ activation is essential for this pathway. PLCβ-triggered intracellular Ca^2+ ^release can lead to the activation of guanine exchange factors (e.g. Sos) for small G proteins like Rac, which then activates JNK. The heavy dependence of JNK on the Ca^2+ ^signal has been previously demonstrated: treatment of Ca^2+ ^chelator BAPTA-AM completely abolished the bradykinin-induced JNK activity in HepG2 cells [32]. Although the PLCβ activation was not completely attenuated by the alanine mutants, diminished flux of intracellular Ca^2+ ^might be already enough to suppress the JNK activity. Several studies from our laboratory have shown that Gβγ complex is a mediator of JNK activation by various GPCRs [32-36], but such regulation is abolished upon suppression of the Ca^2+^-activated SOS/Rac pathway [32,33]. The preservation of ERK activation did not alleviate the suppressive effects of the mutants on JNK activation, which further implied the distinctive regulatory mechanisms of ERK and JNK activation by Gα_16_.

Phosphorylation of NF-κB induced by Gα_16 _was much less affected by the alanine mutations (Figure 3C). Regulation of NF-κB activation is mainly through the IKK/IκB pathway [12], and both Ca^2+ ^and MAPKs play important regulatory roles on IKK/IκB activity. Apparently, PLCβ/Ca^2+ ^signals did not play a dominating role in the NF-κB regulation. The very similar profile of the activation of ERK and NF-κB suggested that ERK might be the key regulator of Gα_16_-mediated NF-κB activation. The essential role of ERK on the regulation of NF-κB has been studied in other cellular contexts [37,38]. The unique interaction of Gα_16 _with TPR1, which promotes the Ras-mediated ERK activation [13], might be one of the possible explanations. Various monomeric G proteins, including Ras and Rac1, can activate NF-κB activity [39].

Mutations of both amino acid clusters resulted in almost complete loss of GPCR-induced PLCβ activation. Consistently, the effect of 3A mutation on the switch III region appeared to be more detrimental than 2A on the α 3 helix, and that seemed to be contrary to the QL mutants. The apparent discrepancies may be due to the different functional impacts of the two mutated clusters towards receptor-mediated activation of Gα_16 _versus the mutation-induced spontaneous activation of Gα_16_QL. It has been shown previously that the intramolecular interactions along the interface between the helical and GTPase domains of Gα_s_, of which switch III is involved, is critical for the receptor-mediated activation of Gα_s _[27,40]. The impact of the mutations of switch III (i.e. 3A mutant) could be revealed when studying the receptor-dependent signaling events, like those in Figures 5 and 6. The results indicated that Gα_16 _made use of similar molecular architecture as Gα_s_, wherein the alterations of the interdomain interactions (3A mutant) exerted a greater effect on the receptor-mediated activation than the mutations in the middle of α 3 helix (2A mutant). However, in the presence of Q212L mutation (the constitutively active mutant), Gα_16 _is simply 'locked' into a GTP-bound state with certain intramolecular conformational adaptations. Such behavior might mask the effect of 3A mutation on the activation process of Gα_16_. Instead, the functional defects caused by the mutations on the exposed surface (2A mutant) became more prominent.

The co-expression of Gα_16 _and GPCRs often leads to an elevated basal IP accumulation [4,24], presumably because the spontaneous activity of receptors becomes amplified through the formation of GPCR/Gα_16 _complexes. Agonist-independent constitutive activation of A_1_R [41,42] and A_2A_R [43,44] have been previously demonstrated. Although not much evidence is available to illustrate the constitutive activity of A_2B_R, inverse agonists for A_2B_R have been identified [45]. Expression of the three alanine mutants resulted in the reduction of the basal IP accumulation, with the effect of 5A being the most prominent, indicating that the G protein complex formed by the alanine mutants reversed the constitutive activity of the adenosine receptors. Such hypothesis is not over-speculative, as it has been shown that the affinity of G_s _for β_2_-adrenergic receptor can be increased by replacing 5 Gα_s _residues in the α 3/β5 loop region with the homologous Gα_i2 _residues [17]. Our previous study also showed that Gα_16/z _chimeras exhibit robust constitutive activity when co-expressed with various GPCRs [24], indicating that Gα_16 _could associate with GPCR and transduce spontaneous receptor activity.

AC2 activation assay was employed to study the release of Gβγ complex from coupled G protein after receptor activation (Figure 6). The assay readout is less sensitive to the functional effect of Gα subunits, and hence mostly reflects the efficiency of receptor-mediated Gβγ release from the activated Gα subunit. One of our previous studies [21] showed that mutations on Gα_16 _causing defective Gβγ interaction significantly reduced its ability to stimulate AC2. In this study, mutations of both but not either one of the clusters destroyed the receptor coupling of Gα_16 _to fMLP receptor. Defective functional release of Gβγ upon receptor activation would suggest that the G protein trimer could not transmit the proper conformational changes from activated receptor or it could not even detect the activated receptor. One possibility is that the pre-associated receptor-G protein complex prefers to stay in an inactivated and associated form, which is echoed by the observation of the reduction of the constitutive receptor activities of the adenosine receptors (Figure 6).

## Conclusion

The two clusters of putative PLCβ-interacting residues on Gα_16 _were indeed crucial for both receptor coupling and regulation of various downstream effectors. The two residues on the α 3 helix were more exposed and important for PLCβ activation, whereas the clusters of three residues on the switch III region affected the receptor-mediated activation of Gα_16 _more. Mutations of either clusters suppressed the activation of STAT3 and, to a greater extent, JNK activation but not ERK and NF-κB signaling. The physical associations between Gα_16 _and PLCβ or TPR1 were sensitive to the mutations, and both clusters were both determinative to the interactions. Further studies on the functional impacts of the mutual interactions between Gα_16_, PLCβ and TPR1 may provide more insights on the characteristics of G_16_-mediated signaling events.

## Methods

### Materials

Human TPR1 cDNA was kindly provided by Richard D. Ye (Department of Pharmacology, College of Medicine, University of Illinois, Chicago, IL). Human embryonic kidney 293 (HEK293) and monkey kidney fibroblast COS-7 cells were purchased from American Type Culture Collection (ATCC CRL-1573 and 1651). Restriction endonucleases were from Roche Applied Sciences. DNA purification columns were from Qiagen. DNA modification enzymes, custom primers and cell culture and transfection reagents were from Invitrogen. [^3^H]adenine and chemiluminescence detection kit for Western blotting were from GE Healthcare. [γ-^32^P]ATP and [^3^H]*myo*-inositol was from PerkinElmer Inc. Antibodies against various molecules are from: Cell Signaling Technology (Danvers, MA, USA) for ERK1/2 (Thr^202^/Tyr^204^), STAT3 (Tyr^705^) and NF-κB (Ser^536^) and their total forms; Torrey Pines Biolabs (C-terminus; Houston, TX, USA) and Gramsch Laboratories (N-terminus, custom-made; Schwabhausen, Germany) for Gα_16_; sc-206, Santa Cruz Biotechnology (Santa Cruz, CA, USA) for anti-PLCβ2; anti-FLAG antiserum and anti-FLAG affinity gel were from Sigma (St. Louis, MO, USA). Protein G-agarose and cross-linking reagent dithiobis [succinimidylpropionate] (DSP) were from Pierce Biotechnology (IL, USA). Pertussis toxin was purchased from List Biological Laboratories. All other chemicals were obtained from Sigma.

### Sequence alignment and molecular modeling

Complete amino acid sequence alignment of Gα 's was generated by CLUSTAL X 1.83 [46]. Molecular models were created by SWISS-MODEL web server [47] using the coordinates of the crystal structures of Gα_i1 _[48] and Gα_t1 _[49] in their corresponding heterotrimers retrieved from Protein Data Bank maintained by Research Collaboratory for Structural Bioinformatics [50] as the modeling templates. The model was modified and visualized with UCSF Chimera [51].

### Construction of Gα_16 _Mutants

Polymerase chain reactions (PCR) were performed to construct the mutants. Amplified cDNA fragment was subcloned into pcDNA3 vector, which possessed T7 and SP6 promoter regions for primer attachment in PCR reaction. Human Gα_16_WT and Gα_16_QL [21] were used as a template to amplify mutants. A pair of overlapping sense and anti-sense primers was designed encoding the desired mutations to replace particular amino acids into alanines. Primers are listed (from 5' to 3') with the mismatch nucleotides underlined: 16-2A/S: CTC GCA TTG TTT GCG GCG ATC CTG GAA CTA CCC; 16-2A/AS: TTC CAG GAT CGC CGC AAA CAA TGC GAG GCT; 16-3A/S: GAG GAG AAC GCA GCT GCA AAC CGC ATG AAG GAG; 16-3A/AS: CAT GCG GTT TGC AGC TGC GTT CTC CTC CAG GCA. The corresponding cDNA fragments were amplified using the desired primers with either T7 or SP6 primer by thermal cycling at 94°C (60 sec)/53°C (60 sec)/72°C (90 sec) for 35 cycles in Thermocycler 40 from Stratagene. Subsequently, the two fragments were pooled together as templates and the full-length mutated cDNAs were generated with the flanking (T7 and SP6) primers. The same thermal cycle program was used for second PCR to amplify full-length products. Full-length PCR products were subcloned in *Xba *I site of pcDNA3. DNA sequences of mutants were verified by autosequencing using BigDye^® ^Terminator v3.1 cycle sequencing kit and ABI PRISM^® ^Genetic Analyser (Applied Biosystems) and restrictive enzyme digestion. 5A and 5A-QL were constructed by using 3A and 3A-QL as templates, respectively.

### Cell Culture and Transfection

HEK293 and COS-7 cells were cultured in Earle's modified essential medium (MEM) and Dulbecco's modified Eagle's medium (DMEM), respectively, with 10% fetal bovine serum (FBS; vol/vol), 50 units/ml penicillin and 50 μg/ml streptomycin. Cells were incubated at 37°C in humidified air with 5% CO_2_. At the day before transfection, COS-7 cells were seeded on 12-well plates at a density of 1 × 10^5 ^cells/well. For western blot analysis, HEK293 cells were used instead, wherein 2 × 10^5 ^cells were seeded on 6-well plates. For adenylyl cyclase assay, 4 × 10^5 ^of HEK293 cells were transferred to 12-well plates. For c-Jun N-terminal kinase assay, 3 × 10^5 ^of COS-7 cells were seeded on 6-well plates. cDNA transfection was achieved using Lipofectamine™ and PLUS™ reagents following the manufacturer's protocol. 50–75% of the cell population will take up the cDNAs as indicated by co-expressing β-galactosidase as a reporter.

### Inositol Phosphate (IP) Accumulation Assay

750 μl of inositol-free DMEM containing 5% FBS and 2.5 μCi/ml *myo*-[^3^H]-inositol was added to each well of transfected COS-7 cells and incubated for 18–24 hr. The labeling media were subsequently replaced by 1 ml of assay medium (DMEM with 20 mM Hepes, pH 7.5 and 10 mM LiCl) for 10 min and then 1 ml of assay medium containing the appropriate agonist was added to the cells for another 1 h at 37°C. Reaction was stopped by adding 750 μl of ice-cold 20 mM formic acid after aspiration and the plates were stored at 4°C for 30 min. [^3^H]-IP were separated from labeled inositol by ion-exchange chromatography as described previously [52].

### cAMP Accumulation Assay

HEK293 cell were labeled with [^3^H]-adenine (1 μCi/ml) in MEM containing 1% FBS (vol/vol) one day after transfection. After 18–24 h the labeling media were replaced by 1 ml of 20 mM Hepes-buffered MEM containing 1 mM isobutylmethylxanthine (IBMX) and the appropriate drugs and incubated at 37°C for 30 min. The treatment was terminated with 1 ml ice-cold 5% trichloroacetic acid (wt/vol) with 1 mM ATP after aspiration and stored at 4°C for 30 min. [^3^H]cAMP was extracted from the pool of labeled nucleotides by sequential ion-exchange chromatography as described [53].

### Data Analysis

For cAMP and IP accumulation assay, absolute values for cAMP or IP accumulations varied between experiments, but variability within a given experiment was in general <10%. The cAMP levels were interpreted as the ratios of the counts per minute of [^3^H]cAMP fractions to those of the total labeled nucleotide fractions and expressed as cAMP/(cAMP + Total). Similarly, IP levels were expressed as IP/(IP + Total). Data shown in the figures were the mean ± S.D. of triplicates within one single experiment. At least three individual experiments yielded similar results. One-way ANOVA with Tukey-Kramer's test were performed using GraphPad Prism 3.03 to verify the significance between different treatment groups within the experiments.

### Cross-linking and Coimmunoprecipitation

Transfected HEK293 cells were washed with PBS twice and then treated in the same buffer containing 0.5 mM DSP for 15 min at room temperature to cross-link the membrane proteins. Cells were washed as above and maintained in quenching solution (50 mM glycine in PBS, pH 7.4) for 5 min, and then lysed in RIPA buffer (25 mM HEPES, pH 7.5, 0.1% SDS, 1% Nonidet P-40, 0.5% sodium deoxycholate, 1 mM dithiothreitol, 200 μM Na_3_VO_4_, 0.7 μg/ml pepstatin, 4 μg/ml aprotinin, 100 μM PMSF, and 2 μg/ml leupeptin). For coimmunoprecipitation, cell lysates were incubated with anti-Gα_16 _(4 μg/sample) or anti-PLCβ2 antiserum (0.4 μg/sample), followed by the incubation with 30 μl of protein G-agarose (50% slurry) at 4°C for 2 h, or 30 μl anti-FLAG affinity agarose gel (50% slurry) at 4°C overnight. Immunoprecipitates were washed by 400 μl RIPA buffer 4 times, and then resuspended in 50 μl RIPA buffer gel loading buffer and boiled for 5 min. Gα_16 _and FLAG-TPR1 proteins in the immunoprecipitates were detected by specific primary antisera and horseradish peroxidase-conjugated secondary antisera using Western blotting analysis.

### Western Blotting Analysis

COS-7 cells were grown on 100-mm dishes to 70–80% confluence. Transfection was performed as in 12-well plates with proper adjustments to the surface area of the plate and the amounts of the reagents used. After 48 h in normal growth conditions, cells were washed with Ca^2+^/Mg^2+^-free Dulbecco's PBS (DPBS) and harvested with 3 ml DPBS containing 10 mM EDTA. The following procedures were performed at 4°C. Cells were spun down briefly (200 *g*, 5 min), resuspended in hypotonic lysis buffer and lysed by one cycle of freeze-thawing followed by 10 passages through a 27-gauge needle. Nuclei were removed by brief spinning and membranes were collected by spinning the supernatants at 15,000 *g *for 15 min. Membrane pellets were finally resuspended in lysis buffer. Detergent-compatible protein assay system (Bio-Rad) was employed to determine the protein contents in cell lysates or crude membrane preparations. Protein samples were separated by 10% SDS-PAGE. Separated proteins were transferred to nitrocellulose membrane by electroblotting. Antisera against specific proteins were applied and the detection was facilitated by ECL chemiluminescence reagents (GE Healthcare). Band intensities on the autoradiographs were analyzed and quantified using ImageJ 1.40 [54].

### *In vitro *c-Jun N-terminal Kinase (JNK) Assay

Details of the assays were basically identical as described in our previous study [55]. Briefly, COS-7 cell were transfected with the appropriate constructs and serum-starved overnight before assay. Cells were lysed by chilled detergent-containing lysis buffer with agitation on ice. 50 μl of each supernatant was used for the detection of JNK-HA expression after removing the cell debris by centrifugation, and the remaining was incubated for 1 h at 4°C with anti-HA antibody (2 μg/sample), followed by incubation with 30 μl of protein A-agarose at 4°C for 1 h. Immunoprecipitates were washed twice with lysis buffer and twice with kinase assay buffer, resuspended in kinase assay buffer with 5 μg of GST-c-Jun, and the kinase reactions were initiated by the addition of 10 μl of ATP buffer (50 μM ATP containing 2 μCi of [γ-^32^P]-ATP per sample). After 30 min incubation at 30°C with occasional shaking, the reactions were terminated by adding sample buffer, and the samples were resolved by 12% SDS-PAGE. The gel was fixed for 30 min and the radioactivity incorporated to GST-c-Jun was detected and quantified by PhosphorImager (Molecular Dynamics 445 SI).

## Abbreviations

A_1_R, A_2A_R, A_2B_R: types 1, 2A and 2B adenosine receptor; AC: adenylyl cyclase; CHA: N^6^-cyclohexyladenosine; D_1_R: type 1 dopamine receptor; ERK: extracellular signal-regulated kinases 1 and 2; fMLP: N-formyl-methioinyl-leucyl-phenylalanine; GPCR: G protein coupled receptor; GTPase: GTP hydrolase; IBMX: isobutylmethylxanthine; IKK: inhibitor of κB kinase; IP: inositol phosphates; JNK: c-jun N-terminal kinase; MAPK: mitogen-activating protein kinase; MEK: MAPK/ERK kinase; MEM: Eagle's minimal essential medium; MKK: MAPK kinase; NF-κB: nuclear factor κB; PKC: protein kinase C; PLCβ: phospholipase Cβ isoform; PTX: pertussis toxin; STAT3: type 3 signal transducers and activator of transcription protein; TPR1: tetratricopeptide repeat protein 1.

## Competing interests

The authors declare that they have no competing interests.

## Authors' contributions

MY carried out most of the experiments, except the co-immunoprecipitation, which was performed by AMFL. MKCH helped to design the mutant constructs, performed molecular model constructions, analyzed and interpreted the results, and drafted the manuscript. YHW conceived of the study and participated in its design and coordination.
